# Analysis of the mechanisms regulating soluble PD-1 production and function in human NK cells

**DOI:** 10.3389/fimmu.2023.1229341

**Published:** 2023-08-10

**Authors:** Francesca Romana Mariotti, Tiziano Ingegnere, Nadine Landolina, Paola Vacca, Enrico Munari, Lorenzo Moretta

**Affiliations:** ^1^ Tumor Immunology Unit, Bambino Gesù Children’s Hospital, IRCCS, Rome, Italy; ^2^ Lymphoid Cells of Innate Immunity Unit, Bambino Gesù Children’s Hospital, IRCCS, Rome, Italy; ^3^ Pathology Unit, Department of Molecular and Translational Medicine, University of Brescia, Brescia, Italy

**Keywords:** NK cells, soluble PD-1, cancer, neuroblastoma, immunotherapy

## Abstract

NK cells represent important effectors that play a major role in innate defences against pathogens and display potent cytolytic activity against tumor cells. An array of surface receptors finely regulate their function and inhibitory checkpoints, such as PD-1, can dampen the immune response inducing an immunosuppressive state. Indeed, PD-1 expression in human NK cells correlated with impaired effector function and tumor immune evasion. Importantly, blockade of the PD-1/PD-L1 axis has been shown to reverse NK cell exhaustion and increase their cytotoxicity. Recently, soluble counterparts of checkpoint receptors, such as soluble PD-1 (sPD-1), are rising high interest due to their biological activity and ability to modulate immune responses. It has been widely demonstrated that sPD-1 can modulate T cell effector functions and tumor growth. Tumor-infiltrating T cells are considered the main source of circulating sPD-1. In addition, recently, also stimulated macrophages have been demonstrated to release sPD-1. However, no data are present on the role of sPD-1 in the context of other innate immune cell subsets and therefore this study is aimed to unveil the effect of sPD-1 on human NK cell function. We produced the recombinant sPD-1 protein and demonstrated that it binds PD-L1 and that its presence results in increased NK cell cytotoxicity. Notably, we also identified a pathway regulating endogenous sPD-1 synthesis and release in human NK cells. Secreted endogenous sPD-1, retained its biological function and could modulate NK cell effector function. Overall, these data reveal a pivotal role of sPD-1 in regulating NK-mediated innate immune responses.

## Introduction

Natural Killer (NK) cells are potent immune effector cells that play a major role in innate defences against viruses and tumors (1). Through an array of inhibitory and activating surface receptors, able to recognize specific ligands induced by virus infection or tumor transformation, NK cells are able to discriminate between healthy and neoplastic cells (2, 3). Indeed, while on one side activating receptors allow recognition and killing of tumor cells, on the other side inhibitory receptors, recognize the HLA class-I molecule expressed on healthy cells preventing their killing and counterbalancing NK cell activation (4–6). However, during cancer progression, the transformed cells might decrease or even lose MHC expression increasing their susceptibility to NK cell-mediated killing. In this context, NK cells are considered powerful weapons against tumors characterized by a very low or absent expression of HLA-I, such as Neuroblastoma (NB) (7–9). Nevertheless, neoplastic cells have developed different mechanisms which sharply dampen NK cell anti-tumour activity. In this context, inhibitory checkpoints have been shown to play a pivotal role in regulating the immune response. Their interactions with specific ligands, often overexpressed or expressed *de novo* by transformed cells, activates a signaling cascade that can dampen NK cell effector function thus promoting tumor growth. One of the major inhibitory checkpoints is represented by the Programmed Cell Death-1 (PD-1) protein that specifically interacts with Programmed Cell Death Ligand 1 and 2 (PD-L1, PD-L2) that have been found to be expressed on different tumors, including NB (10). Several studies have demonstrated that PD-1 can be detected on human NK cells and this expression is associated with impaired NK cell function (11–14). Of note, the PD-1/PD-L1 axis represents a mechanism widely adopted by tumor cells to escape the anti-tumor immune control and blockade of this interaction has been shown to recover NK cell function increasing their cytolytic activity (11, 15).

Recently, soluble counterparts of checkpoint receptors and ligands, in particular of PD-1 (sPD-1) and PD-L1 (sPD-L1), are rising high interest because they could positively or negatively regulate the immune response and are considered novel prognostic markers and therapeutic targets (16–18). sPD-1 originates from an alternative splicing event where the Exon3, containing the coding sequence for the transmembrane domain of PD-1, is deleted from the mRNA transcript (19). Thus, the ΔExon3 isoform, lacking the sequence determining membrane localization, encodes for the soluble form of PD-1. Even though it was reported that no soluble form of sPD-1 could be detected in freshly isolated PBMC from HD, it has been suggested that tumor-specific T cells might be the prime source of circulating sPD-1. While it has recently demonstrated that stimulated macrophages are also able to express sPD-1, no data are available on sPD-1 production by other cell subsets involved in anti-tumor response (20). Of note sPD-1, comprising the extracellular domain required for ligands binding, retains the biological function of the full length PD-1 protein. Accordingly, it is able to interact with PD-1 ligands and thus, blocking their interaction with membrane PD-1 it can regulate the immune response (21). Moreover, few studies indicate that prolonged engagement of PD-Ls by sPD-1 can deliver a reverse signaling altering cell functions (22, 23).

Several *in vitro* and *in vivo* studies on mouse models, aimed to investigate the anti-cancer effect of sPD-1, demonstrated that sPD-1 blockade of PD-L1 could increase activation and cytotoxicity of T cells as well as promote reduction of tumor growth (24–27). Similarly, enhancement of anti-tumor immunity was observed when sPD-1 was combined with gene-therapeutic agents further confirming the broad and pivotal anti-cancer properties of sPD-1 (28, 29). In addition, it has been demonstrated that release of sPD-1 strengthen CAR-T cytotoxicity against CD19^+^ pediatric acute lymphoblastic leukemia cell line and breast cancer cells further demonstrating how sPD-1 could represent a successful therapeutic strategy (30, 31). Importantly, soluble checkpoints are also considered novel therapeutic targets and potential biomarkers. Indeed, high levels of both sPD-1 and sPD-L1 forms have been detected in plasma/serum of different cancer patients, compared to healthy donors (HDs), and have been associated with tumor prognosis, therapeutic response and overall survival (OS) (32–37). Although, several studies demonstrated the pivotal role of sPD-1 in controlling cytotoxic T cells anti-cancer function, there is a lack of knowledge on the role of sPD-1 toward human NK cells. Therefore, in this study we aimed to investigate, on one side whether human NK cell function could be affected by sPD-1 and, on the other side, the mechanisms regulating its production and release.

## Materials and methods

### Human samples

Buffy coats were collected from healthy donors (HD) admitted to the blood transfusion service of IRCCS Bambino Gesù Children’s Hospital after obtaining informed consent. The Ethical Committee of IRCCS Bambino Gesù Children’s Hospital approved (AIRC IG2017#19920) and conducted the study in accordance with the tenants of the Declaration of Helsinki.

### Cells lines

Human neuroblastoma SKNAS and IMR-32 cell lines were purchased from American Type Culture Collection (ATCC, Rockville, MDA) and were cultured in Dulbecco’s Modified Eagle Medium high glucose (Euroclone MI, IT) supplemented with 2 mM l-glutamine (Euroclone), 1% penicillin-streptomycin-neomycin mixture (Euroclone) and 10% heat-inactivated Fetal bovine Serum (FBS) (Euroclone). To detect PD-L1expression, SKNAS and IMR32 cell lines were incubated with anti-PD-L1-PeCF594 (BD Erembodegem, Belgium) and anti-PD-L2-PE-Vio615 (Miltenyi Biotec, Bergisch Gladbach, Germany) for 30 min at 4°C. After wash, samples were acquired using the Cytoflex S (Beckman Coulter) flow cytometer and analysed with the CytExpert 2.4 software (Beckman Coulter, Brea, CA, USA) and the Kaluza software (Beckman Coulter, Brea, CA, USA).

### Isolation and stimulation of NK cells

PBMC were obtained after density gradient centrifugation over Ficoll Lumpholyte^®^-H (Cederlane, Burlington Canada). Highly purified (≥ 95%) CD56^+^, CD3^-^ Peripheral Blood NK (PB-NK) cells were isolated with the Rosette Sep and purity of isolated NK cells was verified incubating NK cells with CD-56-PeCy7 and CD3-APC antibodies. For PD-1 induction, NK cells were stimulated for six days with a cocktail of cytokines consisting of IL-12 (10 ng/ml), IL-15 (25 ng/ml), IL-18 (100 ng/ml) (all purchased from Myltenyi) and DMSO (1:2000; SIGMA) (control NK cells) or 1mM Dexamethasone (Stimulated NK cells) (1:2000, SIGMA) (stimulated NK cells). To verify PD-1 expression, NK cells were stained with CD56-Pe-Cy7 and PD-1-PE (130-117-384, Miltenyi) mAbs 30 min 4°C and acquired using the Cytoflex S (Beckman Coulter) flow cytometer and analysed with the CytExpert 2.4 software.

### Analysis of cytotoxic activity, degranulation and IFN-γ accumulation

For cytotoxicity analysis, control and stimulated NK cells were incubated with SKNAS or IMR-32 cell lines at an effector-to-target (E:T) ratio ranging from 40:1 to 0.3:1. For analysis of both recombinant and endogenous sPD-1, target cells were previously treated with sPD-1 or conditioned media for 30 min at 37°C and then incubated with NK cells. Cytotoxicity was assessed using a flow cytometric assay for NK-cell killing developed by McGinnes (38) and modified as follow: target cells were stained with 5uM of Cell Tracker Green (CMFDA, Invitrogen) for 15 min at 37C. After wash, target cells were incubated with recombinant sPD-1 at 37C for 30 min and then with the effector NK cells. After 4 hrs incubation propidium iodide (Sigma-Aldrich) was added. Live and dead (Td) target cells were identified as CMFDA^+^ PI^−^ and CMFDA^+^ PI^+^, respectively. Specific lysis was calculated as dead target cells (Td) of target cells cultured with effector cells minus Td of target cells cultured alone. To assess degranulation, NK cells were incubated for 4 hrs with SKNAS cells in the presence or not of sPD-1 at 1:1 Effector/Target (E/T) ratio. Monensin (BD, GolgiStop), Golgi Plug (BD) and CD107a-APC (BD) were added to the cocultures. After incubation, cells were stained for surface markers with the following antibodies: CD56-PeCy7 and PD-1-PE. For IFN-γ accumulation NK cells were incubated with SKNAS target cells for four hours in the presence or not of sPD-1. Monensin (BD, GolgiStop), Golgi Plug (BD) were added to the coculture. After incubation, cells were stained with CD56-PeCy7 and PD-1-PE for 30 min at 4°C. After wash cells were fixed and permeabilized with 1% Formalin and 0.1% of Saponin, respectively and then incubated with IFN-γ APC-eF780 (eBiosciences) for 30 min at RT. All samples were acquired with the Cytoflex S flow cytometer and samples were analysed with the CyteExpert and Kaluza software.

### Protein extract and Western Blot analysis

For protein extraction, NK cells pellet were resuspended in RIPA buffer with 1X Halt Protease and phosphatase inhibitor cocktail (Thermo Fisher Scientific, Waltham, Massachusetts, USA), incubated on ice for 20 min and centrifuged for 15 min at 21.130 g at 4°C. Supernatants were recovered and protein concentration was measured with the BCA assay (Perkin Elmer, Waltham, Massachusetts, USA) according to manufacturer’s instruction. For Western Blotting, protein extracts were fractionated by SDS-page gel electrophoresis and transferred to a PVDF membrane (Ge Healthcare, Little Chalfont, UK). Membrane was initially incubated in TBST with 5% nonfat dry milk (Cell Signaling Technology, Danvers, Massachusetts, USA) with gentle agitation for 60 min, and then with TBST with 3% nonfat dry milk containing the following antibodies: α-PD-1 1:1000 (Abnova, Taipei, Taiwan), α-β-Actin 1:10000 (Sigma-Aldrich), Streptavidin-HRP (NEL 75000 1EA, Perkin Elmer) and anti-mouse-HRP (Cell Signaling Technology). Signals were developed with the ECL prime system (Ge Healthcare) according to manufacturer’s instruction and detected with the Uvitec Mini HD9 technology (Uvitec Ltd, Rugby, UK). Quantifications were performed using the Ninealliance© software (Uvitec).

### mRNA extraction and real-time PCR

To isolate RNA, NK cells pellets were resuspended in Trizol (Ambion). Chloroform was added and after incubation on ice for 10 minutes, samples were centrifuged at 12,000g for 15 min at 4°C. The aqueous phase was removed and RNA was then isolated with the Qiagen RNeasy Plus Micro kit (Qiagen, Hilden, Germany) according to manufacturer’s instruction. cDNAs were synthetized by random priming using the Superscript® IV First-Strand Synthesis System (Invitrogen) according to the manufacturer’s instruction. Quantitative Real-time PCR was performed with the QuantStudio 6 Flex PCR (Applied Biosystems) using the PowerUp SYBR Green Master mix (Applied Biosystems) according to manufacturer’s instruction. Relative quantification of mRNA was determined by the ΔCt method. PD-1ΔExon3 mRNA expression was normalized against ActinB expression. Primers for real time are as follow: PD-1- ΔExon3 F 5’- AGGGTGACAGGGACAATAGG -3’; PD-1- ΔExon3 R 5’- CCATAGTCCACAGAGAACAC -3’; ActB-F 5’-ACCGCGAGAAGATGACCCAGA-3’; ActB-R 5’-GGATAGCACAGCCTGGATAGCAA-3’.

### sPD-1 ELISA

sPD-1 levels were quantified by enzyme –linked immunosorbent assays (ELISA) using the human PD-1 antibody duoset kit (DY1086, R&D Systems) and the DuoSet ELISA Ancillary Reagent kit 2 (DY008, R&D). Plates were coated over night with capture antibody (2 ug/ml). After washes (3 x 300ul with Wash Buffer 1X), 100ul of Reagent Diluent were added to each well and plate was incubated for 1 hr at 700rpm. After a washing step as before, plate incubated with 100ul of sample or standards for 2 hrs with gentle shaking. Calibration curve consisted of 1:2 dilutions of the standard material ranging from 2,5 ng/ml to 0,0097 ng/ml. Plate was subsequently washed and incubated with 100ul of Detection Antibody (200ng/ml) for 2 hrs with gentle shaking. After repeating the washing step, 100ul of Streptavidin-HRP was added to each well. Plate was incubated in the dark for 40 min, washed and then treated with 100ul of Substrate Solution for 20 min. After addition of 50ul of Stop Solution, optical density was measured using the Synergy H1 Reader (Biotek, Winooski, USA). Sample were read at 450 nm and 540 nm wavelengths and to correct optical imperfection reading at 540nm was subtracted to reading at 450 nm. sPD-1 concentrations (pg/ml) were calculated using the four-point-fit calibration curve of standard dilutions.

### Luminex

Analysis of soluble forms of inhibitory checkpoints was performed using the MILLIPLEX MAP kit Human Immuno-Oncology Checkpoint Protein Panel 1 HCKP1-11K (Merck) according to manufacturer’s instruction.

### Cloning

The pEX-A258-sPD-1-Tev-T2A-tGFP plasmid, containing the sequence for the PD-1 ΔExon3 isoform in frame with Twin-Strep Tag, T2A and turbo GFP (tGFP) tags with the HindIII and BamHI restriction sites at the 5’ and 3’ends respectively, was obtained from Eurofins Genomics. Sequence verification was performed after each cloning step and congruence was 100%. After transformation into MAX Efficiency^®^ DH5α (Invitrogen) bacterial cells, colonies were selected on Ampicillin (Sigma) plates and DNA was isolated with the QIAprep Spin Miniprep Kit (27106, Qiagen). DNA samples were restricted with BamHI, HindIII and PvuI (New England Biolabs) and products, along with the 1Kb DNA ladder (N3232S, New England Biolabs) were separated by electrophoresis on a 1% agarose gel. Images were collected using the Uvitec Mini HD9 transilluminator system. The band corresponding to the sequence of interest was isolated from the gel with the MinElute Gel Extraction Kit (28604, Qiagen) according to manufacturer instruction. The eluted DNA samples was cloned into the pcDNA3.1 vector, previously restricted with BamHI and HindIII, using the T4 DNA ligase (M0202S, NEB). After antibiotic selection (Ampicillin), DNA was isolated as described before, restricted with BamHI and HindIII enzymes and separated on 1% agarose gel to confirm efficient cloning of the sequence of interest.

### Recombinant sPD-1 protein expression and purification

BL21 DE3 bacterial cells (Invitrogen) transformed with the pCDNA3.1-sPD-1 plasmid were grown in Terrific Broth (Sigma) over night at 30°C and then stimulated with IPTG 1mM (Promega) for 4 hrs. After centrifugation at 3200g 30min at 4°C, cell pellet was resuspended in 20 ml of Sonication Buffer (Hepes 100mM, NaCl 500mM, DTT 1mM and EDTA 0,1mM) and then sonicated as follow: 40% amplitude with 40 sec on/off for 10 times. Sample was centrifuged at 21000g for 30min at 4°C and sPD-1 protein was then purified with the Strep-Tactin®XT purification column according to manufacturer instructions. The purified sample was transferred into Amicon Ultra-4 10K centrifugal filter devices (Merck), centrifuged at 3000g for 20min at 4°C and washed as follow: 4ml of Storage Buffer (NaCl 330mM, Tris-HCl pH6.8 11mM, EDTA 0,11mM and DTT 1,1 mM) at 3000g for 20 min at 4°C for 8 times. Protein concentration was measured with the BCA assay (Perkin Elmer, Waltham, Massachusetts, USA) according to manufacturer’s instruction.

### Coomassie staining

Purified protein sample was fractionated by SDS-Page gel electrophoresis and detected by Coomassie blu dye staining. After run, gel was incubated with the Fixing solution (50% methanol and 10% acetic acid) for 1hr and then stained (50% methanol, 10% acetic acid and 0.1% Coomassie Brilliant Blue R-250) for 20min with gentle agitation. Gel was incubated in the Distaining solution (40% methanol and 10% acetic acid) replenishing the solution several times until background of the gel was fully distained. Gel was stored in 5% acetic acid solution and images were acquired with the Uvitec Mini HD9 technology.

### sPD-1 and sPD-L1 binding

SKNAS and IMR32 cells were firstly treated with PBS and 1% FBS for 30 min and then incubated with 1μg or 5μg of recombinant sPD-1 for 30min at 37°C. After washing, samples were incubated with Streptavidin-PE (Biolegend) antibody 1:50 or PD-L1 1:50 for 30min at 4°C. To demonstrate the specificity of sPD-1 for PD-L1, SKNAS were initially incubated with IgG1 Isotype control (DDXCH01P-100, Novus Biological) or Atezolizumab (A2004, Selleckchem) for 30 min at 4°C and subsequently with recombinant sPD-1 for 30min at 37°C. After washing, samples were incubated with anti-Streptavidin-PE (Biolegend) antibody for 30min at 4°C. Binding of Atezolizumab to PD-L1 was performed incubating SKNAS with IgG1 Isotype control or Atezolizumab for 30 min at 4°C and then with anti-PDL1 for 30 min at 4°C. SKNAS treated with PBS were used as control. All samples were acquired using the Cytoflex S as described before. For analysis of sPD-1 and sPD-L1 binding SKNAS, Ctrl and Stimulated NK cells were incubated with the conditioned media for 1 hours at 37°C and, after washes, samples were incubated with PD-1 or PD-L1 antibody. Binding of Nivolumab (A2002, Selleckchem) to PD-1 was performed incubating stimulated NK cells with IgG4 control Isotype (DDXCH04P-100, Novus Biological) or Nivolumab for 30 min at 4°C and then with anti-PD1 for 30 min at 4°C. Stimulated NK cells, treated with PBS were used as control.

### Statistical analysis

Statistical analyses were performed using the GraphPad Prism 6.0 (La Jolla, CA, USA) software. Values were expressed as mean ± SEM. P values less than 0.05 were considered statistically significant. * P< 0.05, ** P< 0.01 *** P< 0.001.

## Results

**Figure 1:**
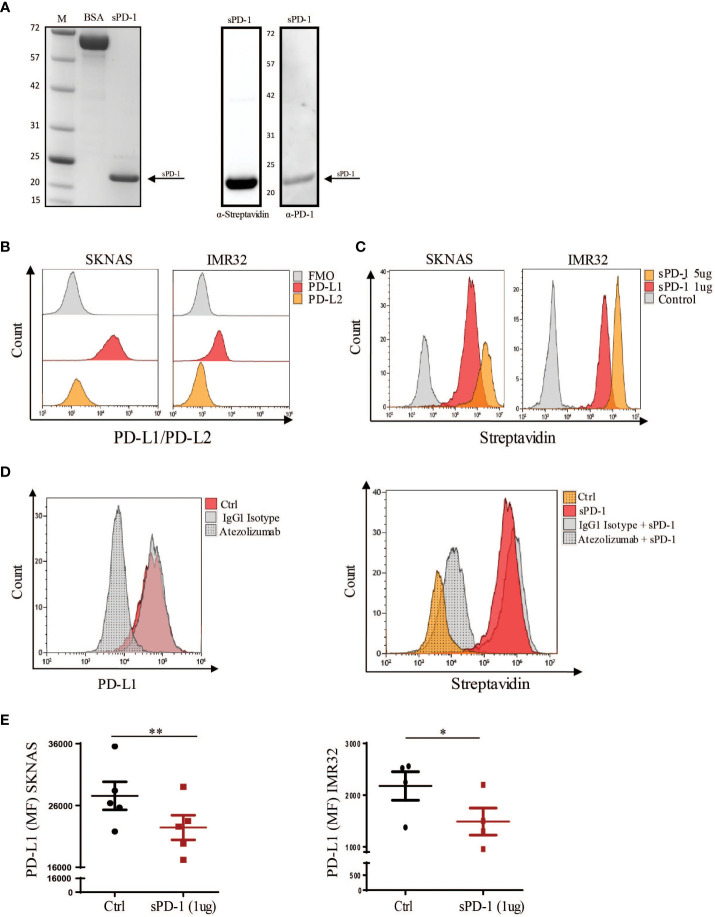
Characterization of the isolated recombinant sPD-1 protein. **(A)** Gel-based proteomic analysis of purified sPD-1. Left panel: Coomassie staining of the isolated recombinant sPD-1 revealed a band of approximately 20 kDa corresponding to sPD-1 molecular weight. BSA sample was used as a control. Right panel: western blot analysis allowed detection of the recombinant sPD-1 protein with both the anti-Streptavidin-HRP and anti-PD-1 antibodies. **(B)** SKNAS and IMR-32 cell lines were analysed for PD-L1 and PD-L2 expression. Only PD-L1 could be detected on the surface of both cell lines. **(C)** Analysis of recombinant sPD-1 binding to PD-L1 ligand. Flow cytometry analysis demonstrated that recombinant sPD-1 was able to bind PD-L1 expressed by SKNAS and IMR-32 cell lines. **(D)** SKNAS were incubated with PBS, IgG1 Isotype or Atezolizumab and then analysed for PD-L1 expression. Abrogation of PD-L1 signal occurred only when tumor cells were treated with Atezoliumab (left panel). Binding of sPD-1 to tumor expressed PD-L1 was abrogated when NB cells were previously incubated with Atezolizumab. **(E)** Incubation of PD-L1^+^ SKNAS and IMR-32 cell lines with recombinant sPD-1 resulted in a decreased PD-L1 signal. MFI from ctrl (not stained) samples was subtracted from their corresponding stained samples. Values are mean ± SEM. Statistical significance has been determined by Paired T test, p< 0.01 **; p<0.05 *.

In the recent years, soluble counterparts of the inhibitory checkpoints, in particular sPD-1 and sPD-L1, have gained attention due to their ability to regulate immune responses. Importantly, these soluble forms retain the ability to bind their ligands and therefore they can inhibit PD-1/PD-DL1 interaction. Indeed, local delivery of sPD-1 in the tumor microenvironment (TME), has been demonstrated to enhance T cell cytotoxicity through PD-L1 binding and the consequent blockade of the PD-1/PD-L1 axis. However, little is known on its biological effect on NK cell function and on the mechanisms regulating its release. Therefore, we decided to investigate whether sPD-1could be able to modulate NK cell effector function. For this purpose, we first synthetized the recombinant sPD-1 protein. To this end, the designed the sPD-1-T2A-tGFP sequence was subcloned into the pcDNA3.1 plasmid and then transformed into the BL21 bacterial cells for protein induction (**Figures S1A, B**). Coomassie staining confirmed that the purified protein was of approximately 20 kDa (**Figure 1A**, left panel), as expected for the recombinant sPD-1 protein. To further evaluate the purity of the isolated protein and also confirm that it effectively corresponded to the soluble counterpart of PD-1 we performed western blot analysis using the α-Streptavidin and α-PD-1 antibodies. As reported in the right panel of **Figure 1A**, hybridization with α-Streptavidin antibody allowed detection of one band corresponding to the isolated recombinant sPD-1 protein. In line with this data, incubation with the α-PD-1 antibody allowed detection of the same band observed with the Streptavidin antibody, thus confirming that the recombinant sPD-1 protein was efficiently purified (**Figure 1A**, right panel). To better characterize the isolated sPD-1 protein, we then investigated whether it retained the functional capability of binding to PD-1 ligands. For this purpose two NB cell lines, SKNAS and IMR-32, that differentially express PD-L1 while having almost undetectable levels of the PD-L2 receptor, were used (**Figure 1B**). The SKNAS and IMR-32 cell lines were incubated alone or with different amounts (1-5 μg) of the recombinant protein and sPD1/PD-L1 interaction was detected as binding of the α-Streptavidin antibody to the Strep-tag present on sPD-1. As reported in **Figure 1C** a shift in the Streptavidin signal could be observed only when sPD-1 was added to the cell cultures, demonstrating that the recombinant protein was able to bind PD-L1 expressed on both cell lines. Interestingly, the intensity of the signal proportionally increased with the amount of sPD-1 (**Figure 1C**). To demonstrate the specificity of sPD-1/PD-L1 interaction, we used Atezolizumab, a fully humanized IgG1 monoclonal antibody (mAb) that engages PD-L1. Indeed, following incubation of SKNAS with Atezolizumab, IgG1 isotype or PBS, abrogation of PD-L1 signal was observed only in the presence of the anti-PD-L1 mAb (**Figure 1D**, left panel). Therefore, to investigate whether mAb treatment would affect PD-L1 engagement by sPD-1, SKNAS were incubated with PBS, Atezolizumab or control IgG1 isotype antibody and subsequently treated with recombinant sPD-1. The interaction between PD-L1 and sPD-1 was analysed by Streptavidin signal and samples treated with PBS represented the negative control. As expected, sPD-1 binding was detected in SKNAS cells treated with PBS and the control isotype while in tumor cells that have been previously treated with Atezolizumab loss of streptavidin signal was detected, demonstrating that masking of PD-L1 prevented sPD-1 binding to its molecular target (**Figure 1D**, right panel). To further investigate the property of sPD-1 binding, the PD-L1 Mean Fluorescent Intensity (MFI) of both SKNAS and IMR32 was analysed. In line with previous data, a partial reduction of PD-L1 MFI was observed in both the NB cell lines incubated with recombinant sPD-1, showing that sPD-1-dependent engagement of PD-L1 could reduce antibody binding (**Figure 1E**). No significant differences regarding PD-L1 MFI were detected upon treatment with 5μg of recombinant sPD-1 (data not shown). Together, these data demonstrate that we efficiently purified the recombinant soluble counterpart of PD-1. Importantly, the isolated sPD-1 protein retained its ability to specifically bind PD-L1 expressed on NB tumor cell lines, as demonstrated by the abrogation of sPD-1/PD-L1 interaction upon treatment with Atezolizumab.

**Figure 2:**
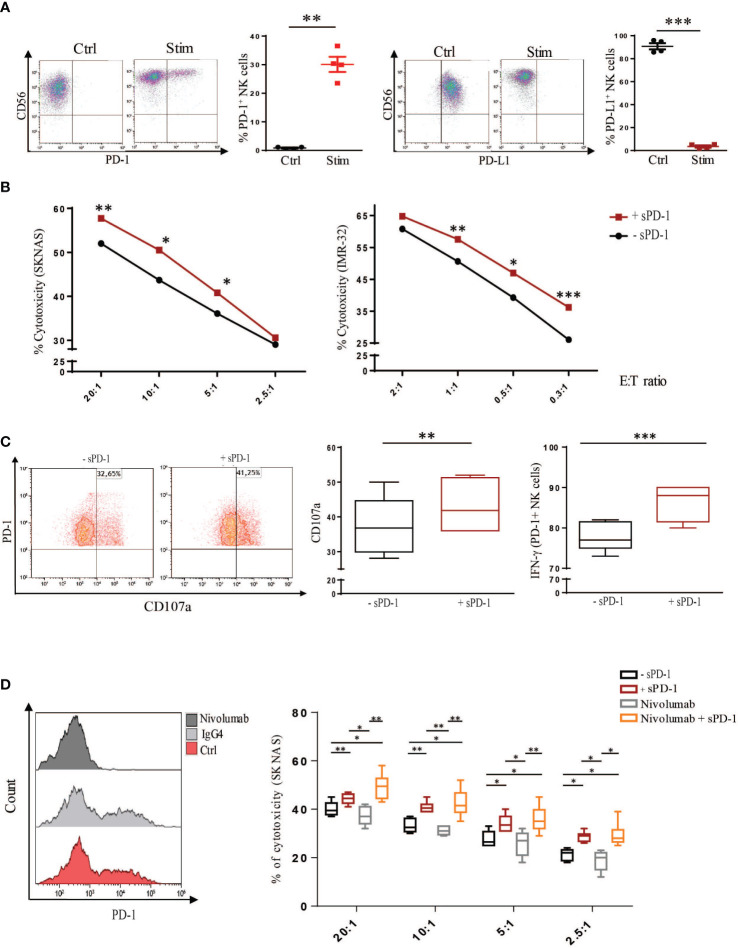
Recombinant sPD-1 can modulate NK cell effector function. **(A)** Control and stimulated NK cells were analysed for membrane PD-1 and PD-L1 expression. Membrane-bound PD-1 was detected only in stimulated NK cells compared to control cells. Conversely, while Ctrl NK cells expressed PD-L1, it was barely detectable on stimulated NK cells. One representative experiment has been shown. Quantifications of four different experiments have been reported. Values are mean ± SEM. Statistical significance has been determined by Paired T test, p< 0.01 **; p< 0.001 ***. **(B)** NB cell lines were incubated with stimulated NK cells, expressing PD-1, with or without recombinant sPD-1. An increase in cytotoxicity toward both SKNAS (n=7) and IMR-32 (n=8) cell lines at different Effector **(E)** Target (T) ratios was observed only when recombinant sPD-1 was present in the co-culture. Data have been compared using paired T test, p< 0.05 *; p< 0.01 **, p< 0.001 ***. **(C)** Similarly, in the presence of recombinant sPD-1 an increase in degranulation (left and middle panels) and accumulation of IFN-γ (right panel) of NK cells were observed. Values are mean ± SEM. Statistical significance has been determined by Paired T test, p< 0.01 **, p< 0.001 ***. **(D)** Comparison of sPD-1 and Nivolumab treatments on cytotoxicity of stimulated NK cells. Incubation with Nivolumab abrogated PD-1 signal compared to Ctrl and IgG4 samples (left panel). Increase of cytotoxicity toward SKNAS cell line at different E:T ratio was observed in both the analysed conditions (treated or not with Nivolumab) only in the presence of sPD-1. Values are mean ± SEM. Data have been compared using paired T test, p< 0.05 *; p< 0.01 **, p< 0.001 ***.

We then asked whether the interaction between sPD-1 and PD-L1, expressed on tumor cells, could affect the ability of NK cells to kill NB target cell lines. For this purpose, freshly isolated NK cells were stimulated *in vitro*, as previously described by our group, with IL-12/IL-15/IL-18 in the presence of DMSO (Ctrl) or Dexamethasone (Stim), in order to induce the expression of PD-1 on the cell membrane (13). Indeed, increase of membrane PD-1 expression was detected in stimulated NK cells compared to control cells (**Figure 2A**). Recently, it has been shown that incubation of NK cells with the same cytokine milieu induces PD-L1 membrane upregulation (39). In line with published data, Ctrl NK cells showed a massive PD-L1 expression (**Figure 2A** left panel). Interestingly, stimulated NK cells do not express PD-L1 at the surface indicating that these receptors are regulated by different mechanisms (**Figure 2A** right panel). Therefore, to investigate any effect of sPD-1 in regulating the immune response we performed the following experiments on Stimulated NK cells that being PD-1^+^ PD-L1^-^ ensure that sPD-1 would bind exclusively to PD-L1 expressed on tumor cells. To analyse the effector functions of stimulated NK cells, cytotoxicity was analysed after 4 hrs incubation with SKNAS or IMR-32 cell lines, in the presence or in the absence of recombinant sPD-1. As reported in **Figure 2B** an increase of NK cell killing was detected, against both NB cell lines, only when sPD-1 was present in the culture medium demonstrating that sPD-1 dependent blockade of PD-1/PD-L1 axis would promote NK cell effector function. In line with these data, increase of degranulation and IFN-γ accumulation were detected in the presence of sPD-1 compared to untreated samples (**Figure 2C** left and right panels).

To further investigate the effect of sPD-1 on the regulation of the immune response, we compared its effect with Nivolumab, an IgG4 humanized mAb able to block membrane PD-1. Indeed, Nivolumab incubation abrogated PD-1 detection on Stimulated NK cells compared to Ctrl and IgG4 isotype-treated cells (**Figure 2D**, left panel). Cytotoxicity was analysed between stimulated NK cells treated or not with Nivolumab in the presence or in the absence of sPD-1. As previously demonstrated, increased tumor cell killing was detected in Stimulated NK cells incubated with sPD-1 compared to ctrl cells (-sPD-1) (**Figure 2D** right panel). Similarly, higher cytotoxicity was detected in Nivolumab-treated cells only in the presence of sPD-1. Interestingly, Nivolumab-treated NK cells exerted an effector function similar to Ctrl cells and it was significantly lower as compared to the same conditions incubated with recombinant sPD-1. No significant differences, except at the higher E:T ratio, could be observed between the two sPD-1 treated samples indicating that, in this condition, Nivolumab treatment does not further improve NK cell killing (**Figure 2D** right panel). These data demonstrate that recombinant sPD-1, through its interaction with PD-L1 expressed by tumor cells, can partially block the inhibitory axis thus improving human NK cell effector function toward NB cell lines.

**Figure 3:**
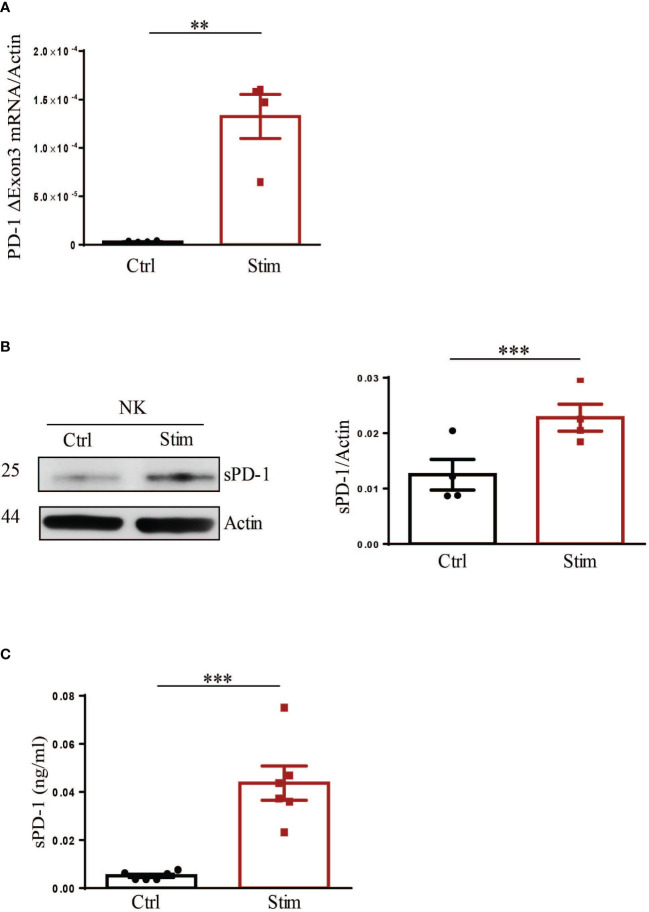
Stimulation of NK cells leads to production and release of endogenous sPD-1. Control (Ctrl) and stimulated (stim) NK cells were analysed for endogenous sPD-1 expression. **(A)** Real-time analysis demonstrated a huge increase of the ΔExon3 mRNA transcript in stimulated compared to ctrl NK cells. **(B)** Western blot analysis demonstrated that an increase in sPD-1 protein level could be observed in NK cells upon stimulation. **(C)** Supernatant of both Ctrl and Stimulated NK cells were analysed by ELISA. High levels of endogenous sPD-1 were detected in the medium derived from stimulated NK cells, while in sample from Ctrl NK cells endogenous sPD-1 could be barely detected. All values are mean ± SEM. Statistical significance has been determined by Paired T test, p< 0.01 **; p< 0.001 ***.

As discussed above, the soluble form of the PD-1 checkpoint inhibitor has been detected in the plasma of cancer patients. However, despite its clinical importance little is known on cell subsets involved in its production and the mechanisms of its release. As mentioned before, our group recently demonstrated that stimulation with a specific combination of cytokines and Dexamethasone, resulted in PD-1 surface expression in human NK cells (13). Therefore, we asked whether the same stimulus could also induce for sPD-1 production and release. To this end, human NK cells, isolated from HDs, were stimulated or not (Ctrl) as previously described and then analysed for sPD-1 synthesis and release. As reported in **Figure 3A**, while the ΔExon3 transcript could be barely detected in Ctrl NK cells, its expression levels were highly increased upon stimulation. In line with this data, sPD-1 protein could be detected only in stimulated NK cells indicating that the increase of the ΔExon3 mRNA transcription correlated with a rise in protein translation (**Figure 3B**). To investigate if the increase in protein translation was associated also to sPD-1 release supernatants from both Ctrl and stimulated NK cells were analysed. As expected, increased sPD-1 levels were detected only in the medium from stimulated NK cells compared to Ctrl samples (**Figure 3C**). All together, these data demonstrate that human NK cells, when properly stimulated, are able to increase both sPD-1 mRNA transcription and protein synthesis as well as its release in the surrounding medium revealing that NK cells may represent a source for circulating sPD-1.

**Figure 4:**
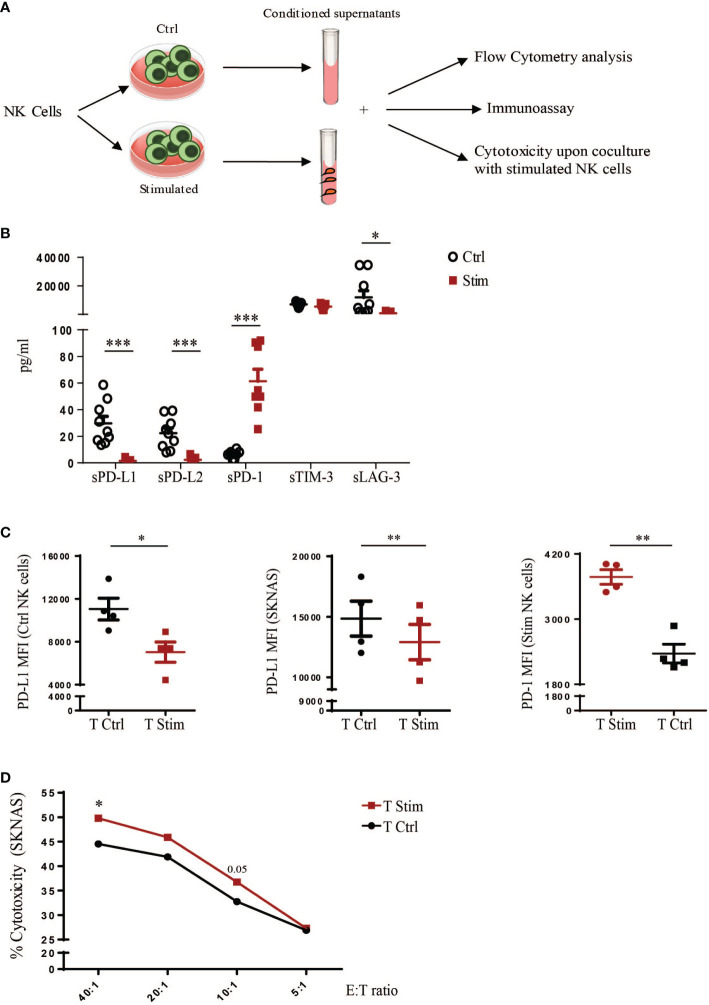
Effects of endogenous sPD-1 on human NK cell function. **(A)** Schematic representation of the designed experiments to investigate the functional activity of endogenous soluble forms released by human NK cells. **(B)** Luminex assay on the SN to evaluate the expression of soluble forms of different inhibitory checkpoints released by human NK cells in the two treatment conditions. All values are mean ± SEM. Statistical significance has been determined by Paired T test, p< 0.05 *; p< 0.001 ***. **(C)** Ctrl and Stimulated NK cells and SKNAS cell line were incubated with conditioned medium deriving from unstimulated (Ctrl) or stimulated (Stim) NK cells and binding of endogenous sPD-1, and sPD-Ls present in the medium, was analysed by flow cytometry as decrease in PD-L1 and PD-1 MFI. Values are mean ± SEM. Statistical significance has been determined by Paired T test, p< 0.05 *; p< 0.01 **. **(D)** Cytotoxicity of stimulated NK cells toward SKNAS cell line at the indicated Effector (E): Target (T) ratios upon incubation with conditioned media. Values (% of cytotoxicity) represent the mean of nine independent experiments. Data have been analysed using paired T test, p< 0.05 *.

We then studied whether endogenous sPD-1, produced by NK cells, retained its ability to interact with PD-L1 and whether this engagement would affect NK cell effector function. For this purpose human NK cells were stimulated or not in order to obtain different conditioned supernatants (SN) that were then used to treat tumor cells and study the sPD-1 biological function (**Figure 4A**). Even though we previously demonstrated that sPD-1 could be detected only in the SN from stimulated NK cells we could not exclude that the two conditioned media would differ exclusively for sPD-1 expression. Therefore, we analysed the expression of soluble forms of different inhibitory checkpoints in the two SN. Interestingly, in the media derived from Ctrl NK cells sPD-L1 and sPD-L2 levels were higher compared to SN from stimulated NK cells (**Figure 4B**). Conversely, sPD-1 was the only soluble form upregulated in SN from stimulated NK cells. Of note, these data are in line with PD-1 and PD-L1 membrane expression on NK cells. To investigate whether endogenous sPD-1 would be able to bind PD-L1, both Ctrl NK cells and SKNAS were incubated with the different SN and PD-L1 expression was analysed. Reduction of PD-L1 MFI was observed in both samples only when incubated with the medium containing sPD-1 (**Figure 4C** left and middle panels). Interestingly, a reduction of PD-1 MFI occurred only in Stimulated NK cells incubated with SN containing sPD-Ls indicating that these soluble forms were able to interact with membrane PD-1 reducing antibody binding (**Figure 4C** right panel). We further investigated whether the interaction between endogenous sPD-1 and PD-L1 could have an effect on NK cell function as previously detected with the recombinant sPD-1 protein. Thus, SKNAS cells and stimulated human NK cells were incubated together using the supernatants from ctrl and stimulated NK cells in order to compare blockade of the PD-1/PD-L1 axis acting on PD-1 or tumor expressing PD-L1, respectively. Except for the higher E:T ratio, where increased cytotoxicity was observed in the presence of SN from stimulated NK cells no significant differences could be observed even though there is a trend of higher cell killing when PD-L1 ligand is bound (**Figure 4D**).

## Discussion

In cancer, the PD-1/PD-L1 axis has been the subject of intense investigations and its blockade, through the development of mAbs disrupting this interaction, has revolutionized tumor immunotherapies. Recently, the soluble counterpart of PD-1 has gained interest due to its prognostic and predictive value in tumor patients, which has opened a new paradigm of investigation in different cancer types. In addition, sPD-1, retaining the function proper of full-length PD-1, is able to engage with membrane-bound ligands and enhance T cell-dependent anti-tumor immune responses limiting the binding of PD-1^+^ cells with PD-Ls^+^ neoplastic cells. In this context, we investigated the role of sPD-1 toward human NK cell function and the mechanisms regulating its expression. In particular, we purified a recombinant form of sPD-1 able to interact with PD-L1 expressed by tumor cell lines. The sPD-1/PD-L1 interaction was specific as demonstrated by the abrogation of sPD-1 binding upon treatment of tumor cells with Atezolizumab and by reduction of PD-L1 MFI indicating that sPD-1might act as a decoy for anti-PD-L1 antibody. Importantly, sPD-1-dependent blockade of PD-L1 pathway was able to modulate the anti-tumor immune response of human NK cells. Indeed, we observed an increase of tumor cell killing, degranulation and IFN-g accumulation in the presence of sPD-1 as compared to ctrl (-sPD-1) samples.

Considering the biological importance of the circulating soluble form of PD-1, we focused our attention on mechanisms regulating endogenous sPD-1 production. In a previous work, investigating the expression of PD-1 in human NK cells, we demonstrated that resting and *in vitro* activated NK cells, isolated from HDs, expressed the PD-1 ΔExon3 mRNA isoform, even though no sPD-1 protein could be detected in the cytoplasm or in the culture supernatants (40). Moreover, sPD-1 was detected in the pleural effusions (PE) of lung cancer patients and the ΔExon3 mRNA transcript was detected in NK cells purified from the same PE. Therefore, we argued that human NK cells could be involved in sPD-1 production. Indeed, we demonstrated that specific stimulation of human NK cells leads to increase sPD-1 transcription, synthesis and release in the culture medium. These data unveil a novel mechanism regulating sPD-1 production and also demonstrates that human NK cells are capable of releasing sPD-1. Of note, analysing the different SN we confirmed that released of sPD-1 would occur only in Stimulated NK cells and showed that ctrl NK cells are able to release sPD-L1 and sPD-L2 in the culture medium indicating that NK cells could be considered a source for sPD-Ls circulating forms.

Interestingly, binding assays showed decreased PD-L1 and PD-1 MFI upon treatment of NK and SKNAS cells with SN from stimulated and ctrl NK cells, respectively indicating that soluble checkpoint inhibitors retained the ability to interact with their specific targets and thus could act as decoy for antibody binding. However, comparing PD-L1 and PD-1 blockade, taking advantage of the different conditioned media, no significant differences could be observed.

Despite our demonstration of the involvement of human NK cells in producing sPD-1 and its role in regulating antitumor immune response, some limitations of this study must be considered. The recombinant sPD-1 protein, being produced in bacteria, does not present the posttranslational modifications (PTMs) proper of full-length PD-1 and known to be important for its function and therefore in our setting we could not investigate how the PTMs would affect recombinant sPD-1 function. In addition, the effects of endogenous soluble forms on NK cell effector function are less marked compared to the modulation observed with the recombinant sPD-1 protein. This could be explained by the fact that the average amount of released sPD-1 and sPD-L1 by stimulated and ctrl human NK cells is quite low and it might not be sufficient to efficiently block the PD-1/PD-L1 pathway and modulate NK cell anti-tumor activity. Indeed, median values of circulating sPD-1 in serum/plasma of cancer patients can span from almost 400 pg to even 8 ng or more (27, 32). Moreover, in the TME, due to the close cellular interactions and to the presence of several cytokines and other soluble factors, the concentrations of sPD-1 may actually be quite higher. Therefore, in the present set up, it is conceivable that we underestimate the real contribution of endogenous sPD-1 on human NK cell function. In this context, it would be interesting to purify the endogenous sPD-1 protein in order to deeper investigate its biological function and define the amount sufficient for enhancing NK-mediated function. Indeed, there is a lack of information in the literature on the role played by sPD-1 in the context of pediatric tumors and the presented data might suggest that sPD-1 could play a role as an immune regulator in NB.

In conclusion, we identified a mechanism regulating sPD-1 synthesis and release in human NK cells demonstrating that this innate cells can also be considered a source for circulating sPD-1. Moreover, our present study provides a first insight on the effect of sPD-1 on human NK cell effector function, confirming that sPD-1, retaining its ability to bind PD-L1, can disrupt the PD-1/PD-L1 axis and counteract the inhibitory effect of this interaction.

Overall, these data confirm the important role played by sPD-1 in regulating the anti-tumor immune response and suggest that sPD-1 could be considered a novel additional tool for anti-cancer therapies in paediatric tumors.

## Data availability statement

The original contributions presented in the study are included in the article/**Supplementary Material**. Further inquiries can be directed to the corresponding author.

## Ethics statement

The studies involving human participants were reviewed and approved by Ethical Committee of Bambino Gesù Children’s Hospital, IRCCS. The patients/participants provided their written informed consent to participate in this study.

## Author contributions

FM designed and performed experiments, interpreted data and wrote the manuscript. TI and NL performed experiments and reviewed the manuscript. EM reviewed the manuscript. PV and LM discussed results and reviewed the manuscript. All authors contributed to the article and approved the submitted version.

## Supplementary material

Supplementary Figure 1 Construction of the pCDNA3.1-sPD-1-Twin-Strep-tag-T2A-GFP plasmid. **(A)** Left panel: schematic representation of the region, within the pEX-A258 plasmid, containing the sPD-1 coding sequence in frame with the TEV site and the Twin-Strep-tGFP sequence. HindIII and BamHI restriction enzyme sequences were introduced at the beginning and the end of the described region, respectively. Right panel: upon DH5alpha bacterial cells transfection, positive colonies were screened by HindIII, BamHI and PvuI restriction and samples were separated on agarose gel. The red arrow indicates the band of 2187 bp corresponding to the sPD-1-Twin-Strep-tGFP region. **(B)** Circular map of the pCDNA3.1-sPD-1-Twin-Strep-tag-T2A-GFP plasmid upon subcloning of the sPD-1-Twin-Strep-tGFP region (left panel). Colonies grown in selective plates were analysed by HindIII and BamHI restriction and samples were run on agarose gel (right panel). The red arrow indicates the sPD-1-Twin-Strep-tGFP band.
