# Supplementary figures and images for: Analysis of the mechanisms regulating soluble PD-1 production and function in human NK cells
Source: Front Immunol. 2023 Aug 10;14:1229341. doi: 10.3389/fimmu.2023.1229341 (PMC10449250; doi:10.3389/fimmu.2023.1229341)

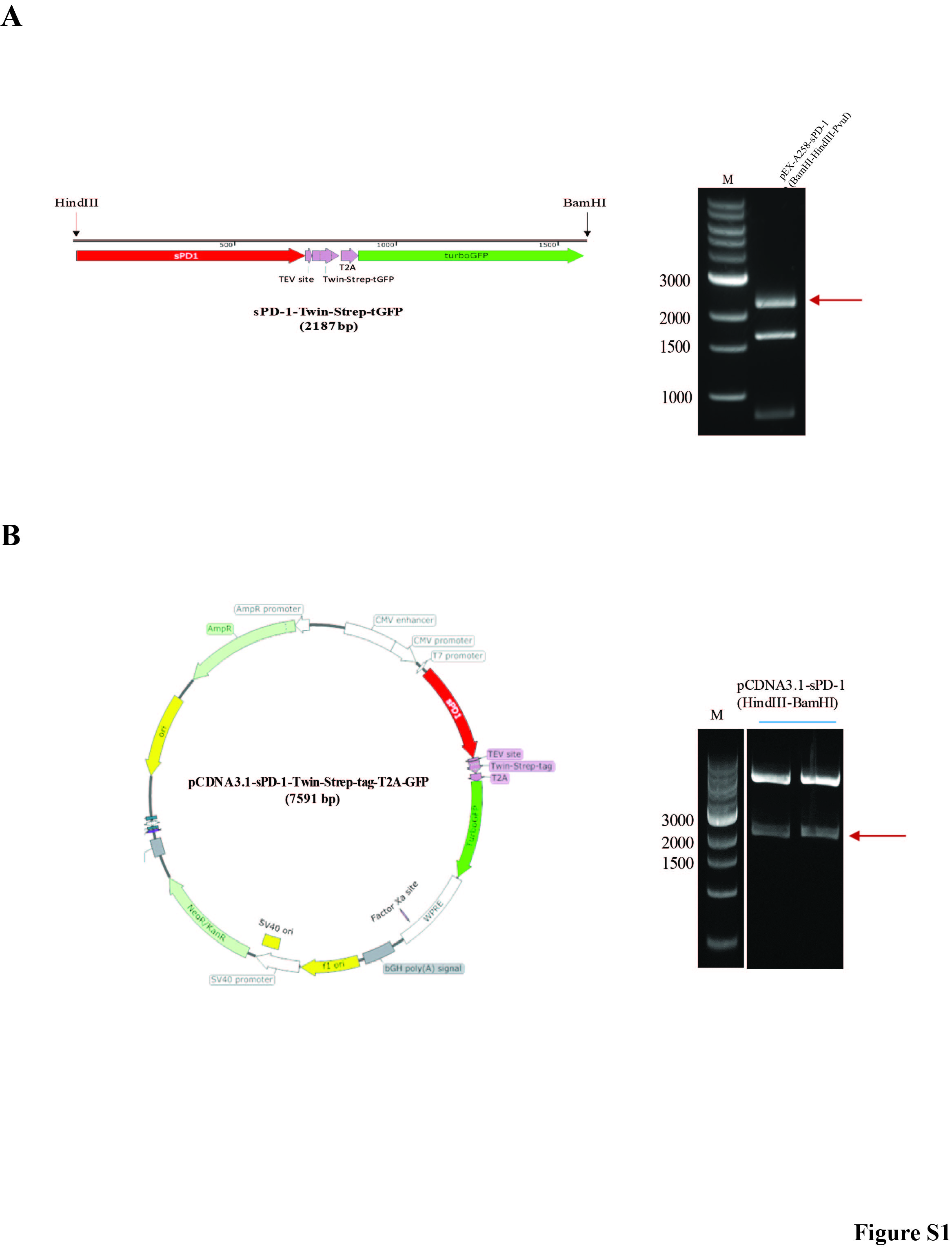

Supplement: Supplementary Figure 1 — Construction of the pCDNA3.1-sPD-1-Twin-Strep-tag-T2A-GFP plasmid. (A) Left panel: schematic representation of the region, within the pEX-A258 plasmid, containing the sPD-1 coding sequence in frame with the TEV site and the Twin-Strep-tGFP sequence. HindIII and BamHI restriction enzyme sequences were introduced at the beginning and the end of the described region, respectively. Right panel: upon DH5alpha bacterial cells transfection, positive colonies were screened by HindIII, BamHI and PvuI restriction and samples were separated on agarose gel. The red arrow indicates the band of 2187 bp corresponding to the sPD-1-Twin-Strep-tGFP region. (B) Circular map of the pCDNA3.1-sPD-1-Twin-Strep-tag-T2A-GFP plasmid upon subcloning of the sPD-1-Twin-Strep-tGFP region (left panel). Colonies grown in selective plates were analysed by HindIII and BamHI restriction and samples were run on agarose gel (right panel). The red arrow indicates the sPD-1-Twin-Strep-tGFP band. [file Image_1.jpeg]
